# Enhancing self-compassion in individuals with visible skin conditions: randomised pilot of the ‘My Changed Body’ self-compassion writing intervention

**DOI:** 10.1080/21642850.2019.1587298

**Published:** 2019-03-18

**Authors:** Kerry A. Sherman, Tegan Roper, Christopher Jon Kilby

**Affiliations:** Department of Psychology, Centre for Emotional Health, Macquarie University, Sydney, Australia

**Keywords:** Self-compassion, therapeutic writing, skin condition, affect, My Changed Body

## Abstract

**Background:** Abnormalities in the appearance of skin are commonly associated with compromised self-body perceptions, arising from physical manifestations of the skin condition that deviate from the individual's idealised body image. These body image concerns are associated with a range of psychological issues including anxiety, depression, fear of negative evaluation, and suicidal ideation. Unfortunately, stigma and embarrassment associated with these body image concerns mean that these issues are rarely discussed in clinical medical consultations. There is thus a need for highly accessible and acceptable interventions to address skin-related body image concerns. We have previously demonstrated that a web-based self-compassion focused therapeutic writing approach, the ‘My Changed Body’ intervention, is efficacious in addressing body image concerns of women in the breast cancer context. The aim of this experimental pilot study was to investigate the feasibility of applying the My Changed Body intervention to address visible skin-related body image concerns.

**Methods:** Participants (*N* = 50) with a range of visible skin conditions provided online informed consent, then completed measures of demographic and medical history, body image disturbance, self-compassion and positive and negative affect. They were then randomly allocated either to an active control expressive writing condition (*n* = 25) or to the My Changed Body writing condition (*n* = 25). Participants were blind to their condition allocation. Immediately after completing their allocated writing exercise, participants completed self-compassion and affect measures.

**Results:** Controlling for pre-writing body image disturbance, repeated measures ANCOVAs with fixed effects revealed that self-compassion and negative affect significantly improved after the My Changed Body writing exercise, compared to the control condition. There was no between groups difference at follow-up in positive affect.

**Conclusions:** This study suggests that the My Changed Body writing intervention may provide benefit to individuals with visible skin conditions. A randomised controlled trial is needed to further confirm these results.

## Introduction

Skin conditions (e.g. eczema and psoriasis) represent the fourth-most common cause of non-fatal disease burden globally, particularly in Western countries such as Australia, the United States of America and Northern Europe (Baker, Foley, & Braue, 2013). In the United Kingdom, almost one in two (54%) will experience some kind of skin condition each year, with up to 25% of primary care consultations focussing on skin conditions (Bundy et al., 2013). The skin, as the largest and most visible organ of the body, is strongly implicated in an individual’s sense of wellbeing and attractiveness (Rumsey, 2018). Stigmatisation based on physical appearance is commonly experienced by individuals with visible skin conditions and can lead to social rejection (Bundy et al., 2013; Ginsburg & Link, 1993), disparaging labels from others (Kellett, 2014), and impaired intimate relationships (Baker et al., 2013; Gupta & Gupta, 1998). Such stigmatisation has been linked to depression, anxiety and low self-esteem (Hong, Koo, & Koo, 2008; Murray & Rhodes, 2005; Picardi, Mazzotti, & Pasquini, 2006). Notably, degree of psychosocial impairment is not directly related to the severity of the skin condition symptoms (Gupta & Gupta, 1998; Law, Chuh, Lee, & Molinari, 2010; Picardi et al., 2006).

Given the highly visible nature of skin disorders, body image is frequently adversely impacted. Body image is defined as an individual’s perceptions, thoughts, feelings, and behaviours associated with their physical appearance (Sarwer & Spitzer, 2015). In the case of individuals with a dermatological condition, the physical manifestations of the skin condition that deviate from the individual’s idealised body image will compromise their sense of self, leading to feelings of body image disturbance (Cash, 2011; Cash & Szymanski, 1995; White, 2000). This skin condition-related disturbance of body image has been associated with increased insomnia (Gupta, Gupta, & Knapp, 2015), anxiety, depression (Hinkley, 2015), fear of negative evaluation, suicidal ideation (Gupta & Gupta, 2013) and diminished self-esteem (Kouris, Platsidaki, Kouskoukis, & Christodoulou, 2017). Reluctance to seek professional help with managing these body image concerns is associated with feelings of embarrassment and shame (Ahmed et al., 2018; Kouris et al., 2017), consistent with research reporting that bodily embarrassment is associated with less frequent medical contact, generally (Consedine, Krivoshekova, & Harris, 2007). Stress, anxiety, negative affect (emotion), and behavioural habits (e.g. scratching) can exacerbate skin conditions leading to flare ups or poor control (Shenefelt, 2010).

A number of psychosocial interventions have been developed to manage skin conditions, sometimes in conjunction with frequently-prescribed pharmacological therapies (Rumsey, 2018). These psychotherapeutic approaches include interventions such as cognitive behavioural treatment (CBT), hypnosis, and biofeedback, all of which tend to be time- and labour-intensive, requiring in-person specialist consultation (Shenefelt, 2010). To date, only CBT has demonstrated promising results (Rumsey, 2018). Online CBT-focused interventions, such as eTIPs (for psoriasis; Bundy et al., 2013) and FaceIT/YPFaceIT (for visible skin conditions in adults and young people respectively; Bessell, 2012; Williamson, Griffiths, & Harcourt, 2015) provide an efficacious and alternative means of accessing psychological support whilst overcoming geographical barriers to accessing in-person trained psychotherapy providers. However, these online interventions have experienced high attrition rates (Rumsey, 2018). Dropout from online interventions, generally, is a common limitation (Karyotaki et al., 2017; Köhle et al., 2015; Richards & Richardson, 2012; Sundström, Blankers, & Khadjesari, 2017) and may be related to intervention users feeling overwhelmed by the time commitment and intensity of material to cover (Beatty et al., 2017; Finlay-Jones, Kane, & Rees, 2017; Kuijpers, Groen, Aaronson, & van Harten, 2013; Postel, de Haan, Ter Huurne, Becker, & de Jong, 2010). Briefer, less intensive online interventions potentially provide a useful alternative approach to reach those individuals lacking in the time or motivation to engage with more complex online interventions. There is, therefore, a need for a brief and readily accessible online intervention to address body image concerns associated with skin conditions that are easy to use and acceptable to this population, as left untreated these difficulties may contribute to further psychological distress and compromised quality of life (Ahmed et al., 2018).

One emerging therapeutic approach to managing body-image concerns is self-compassion, a kind and gentle manner of relating to oneself during suffering, comprising self-kindness, common humanity, and mindfulness (Neff, 2003; Neff & Germer, 2017). Individuals who adopt a self-compassionate perspective in the face of distress or failure tend to treat themselves with warmth and understanding, rather than criticism (Neff, 2003; Neff et al., 2018). Research in healthy student and community populations has found that higher self-compassion is associated with greater psychological well-being, less psychological distress (MacBeth & Gumley, 2012; Van Dam, Sheppard, Forsyth, & Earleywine, 2011), positive affect (Neff et al., 2018), positive coping, and fewer maladaptive coping strategies (Allen & Leary, 2010; Neff & Dahm, 2015; Pinto-Gouveia, Duarte, Matos, & Fráguas, 2014). The proposed mechanism is through emotion regulation, whereby self-compassion enables an individual to more effectively regulate emotions when facing threats and stressors, in turn reducing their impact (Finlay-Jones et al., 2017). Empirical evidence supports this view with self-compassion being negatively associated with emotion regulation strategies that are linked with psychological distress, such as rumination (Krieger, Altenstein, Baettig, Doerig, & Holtforth, 2013; Neff, 2003; Raes, 2010), thought suppression (Neff, 2003; Neff, Kirkpatrick, & Rude, 2007), and avoidance (Costa & Pinto-Gouveia, 2013; Finlay-Jones et al., 2017; Krieger et al., 2013; Roemer et al., 2009). Self-compassion is also negatively associated with problems recognising, understanding, or accepting certain emotional states; and difficulties accessing effective coping strategies, controlling impulsive behaviour, and maintaining goal-directed behaviour when experiencing emotional discomfort (see Gratz & Roemer, 2004). In further support of the emotional regulation view, there is growing evidence that emotion regulation difficulties mediate the link between self-compassion and psychological distress, including stress (Finlay-Jones, Rees, Kane, & van der Feltz-Cornelis, 2015), depression (Krieger et al., 2013; Raes, 2010), and anxiety (Raes, 2010).

In an illness context, higher self-compassion has been associated with lower negative affect, anxiety and depression (i.e. influenza, asthma, cancer), lower stress (i.e. HIV+, chronic illness, cancer), and greater quality of life (Pinto-Gouveia et al., 2014). In the cancer context, there is evidence that individuals with low self-compassion are more likely to experience difficulties in romantic interpersonal relationships (Shaw, Sherman, Fitness, Elder, & Breast Cancer Network Australia, 2018) which are related to ongoing body image disturbance (Przezdziecki et al., 2013). There is also evidence from clinical populations of individuals with body image concerns arising from eating disorders and following physical illness, including cancer treatment, that greater self-compassion is associated with less psychological pathology and body image distress (Braun, Park, & Gorin, 2016; Duarte, Ferreira, Trindade, & Pinto-Gouveia, 2015; Przezdziecki et al., 2013; Van Dam et al., 2011). Taken together, this evidence indicates that self-compassion is a key factor in adjustment to challenging experiences, such as chronic and serious health conditions (Pinto-Gouveia et al., 2014; Sherman et al., 2018), and may be a helpful emotion regulation strategy in which to address body image difficulties associated with skin conditions by encouraging willingness to experience negative emotions, and accepting them as part of human experience (Neff, 2003; Odou & Brinker, 2015; Rodgers et al., 2018).

Self-compassion has predominantly been conceptualised as a trait, however, targeted interventions in healthy (Arch et al., 2014; Finlay-Jones et al., 2017; Neff & Germer, 2013) and physical illness (Braun et al., 2016; Sherman et al., 2018) populations have demonstrated its modifiable and trainable nature. It has been proposed that self-compassion training acts by promoting emotion regulation (Arch et al., 2014) and coping in the face of difficult experiences (Allen & Leary, 2010). In physically healthy adolescent populations with body image concerns, self-compassion focused interventions entailing meditations (Albertson, Neff, & Dill-Shackleford, 2015) and weekly training on being non-judgemental (Atkinson & Wade, 2015, 2016), demonstrated reductions in body image disturbance and body shame, and improvements in body appreciation. Given the reluctance for individuals with skin condition-related body image concerns to seek psychotherapy, and the advantages in accessibility and dissemination, internet-based interventions provide a viable means through which to deliver therapeutic interventions for this populations. Internet-based interventions, generally, have demonstrated efficacy in addressing body image difficulties. A review of 20 digital health interventions addressing eating disorder symptoms in adolescents and young adults, reported that compared with control conditions, internet-based interventions led to significant improvements in body image and reductions in negative affect (Melioli et al., 2016). Further, another digital health intervention addressing body image concerns in adolescents, BodiMojo, specifically applied a self-compassion focused therapeutic approach through a mobile application (Rodgers et al., 2018). Evidence from a randomised controlled trial of BodiMojo provides preliminary evidence that this intervention led to improvements in self-compassion and appearance-related esteem (an indicator of body image). Thus, there is a growing body of evidence supporting both the use of self-compassion focused interventions for promoting body image (Braun et al., 2016) and for digital interventions (Melioli et al., 2016; Rodgers et al., 2018) to provide benefit in this context.

One promising intervention approach designed to enhance self-compassion, and thereby potentially address psychological concerns is through self-compassion focused therapeutic expressive writing (Leary, Tate, Adams, Batts Allen, & Hancock, 2007; Sherman et al., 2018). Expressive writing is a form of emotional expression in which a person writes about a major life event or distressing concern, without regard for audience, form or other writing conventions (Merz, Fox & Malcarne, 2014). This type of expressive writing has been demonstrated through meta-analysis to improve functioning in a range of domains including self-reported physiological health, psychological health and general functioning (Smyth, 1998). Integrating the advantages of a digital intervention approach for addressing body image concerns (Melioli et al., 2016; Rodgers et al., 2018), we have developed a web-based therapeutic writing intervention known as ‘My Changed Body’ (MyCB), that modifies the concept of therapeutic expressive writing about one’s deepest thoughts and emotions (Pennebaker, 1997; Stanton et al., 2002), by providing guided prompts with a focus on self-compassion (Przezdziecki, Alcorso, & Sherman, 2016), similar to that developed by Leary et al. (2007). The MyCB intervention aims to encourage the writer to perceive negative and distressing experiences in a supportive and caring manner (Sherman et al., 2018). This brief, one-session intervention was originally developed to address body image concerns and associated psychological distress of women recovering from breast cancer surgery and treatment (Przezdziecki et al., 2016). Evidence from a large longitudinal randomised controlled trial demonstrated that, compared with expressive writing controls, breast cancer survivors undertaking the self-compassion focused MyCB writing reported significantly less body image distress and enhanced perceptions of their body image up to three months following the writing intervention (Sherman et al., 2018). Moreover, these improvements were demonstrated to be mediated by enhancements in self-compassion at one week following exposure to the MyCB writing intervention (Sherman et al., 2018), which in turn impacted the body image perceptions. Notably, the subset of participants in this study who also had a diagnosis of lymphoedema (a disabling and highly visible side-effect of cancer treatment) and who experienced the greatest body image-related distress at study entry, experienced significant reductions in both depression and anxiety up to three months following completion of the MyCB self-compassion writing, compared with controls (Sherman et al., 2018).

As a low-cost alternative to group-administered programmes, the MyCB web-based intervention provides a time-efficient, low-burden (Przezdziecki et al., 2016) therapeutic approach for addressing body image concerns. Since MyCB is based on enhancement of self-compassion through structured writing concerning negative experiences related to bodily disfigurement or negative changes, this therapeutic methodology is eminently translatable to other physical illness contexts. The benefit of writing about personal distress appears to be greatest in those who belong to a stigmatised group (Richards, Beal, Seagal, & Pennebaker, 2000), such as individuals with a visible skin condition. Moreover, a more time-intensive self-compassion-based training intervention has demonstrated reductions in shame and improved psychosocial and emotional functioning among people with acne (Kelly, Zuroff, & Shapira, 2009). Therefore, in light of the ever-increasing access of the general Australian population to the internet (Australian Bureau of Statistics, 2018), which is estimated to be more than 85%, the web-based MyCB intervention may provide a viable way to support individuals with visible skin conditions experiencing body image concerns. The aim of this study was to conduct a pilot trial of the MyCB intervention to assess feasibility of this therapeutic approach being translated to the visible skin condition context. We therefore hypothesised that the MyCB intervention would enhance self-compassion and modify emotions such that there would be a significant decrease in negative affect and increase in positive affect, compared to the active control condition consisting of an expressive writing exercise without self-compassion based writing prompts. By demonstrating that the MyCB intervention can produce immediate shifts in self-compassion and affect, mimicking prior work using this intervention (Przezdziecki & Sherman, 2016), then this would justify a larger RCT to further assess the utility of the MyCB intervention in this population.

## Methods

### Participants

**Figure 1.**
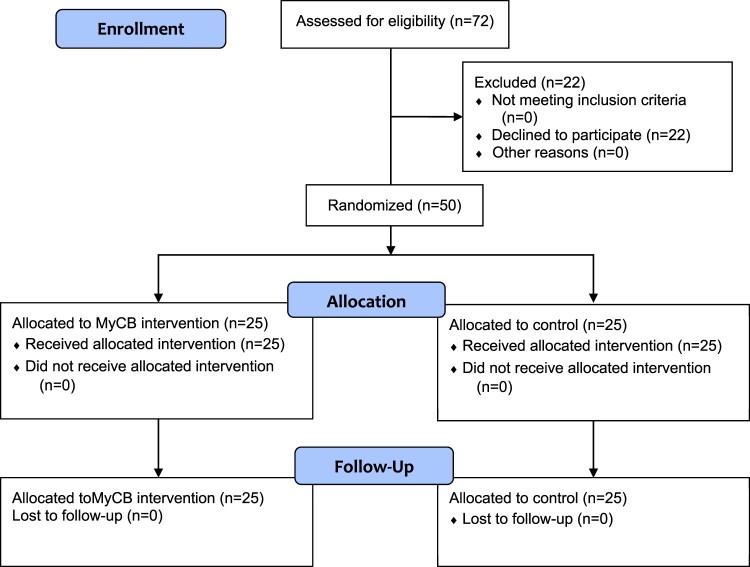
CONSORT randomised pilot diagram.

Individuals over 18 years who were experiencing a currently symptomatic and visible skin condition (e.g. eczema, psoriasis, acne) and who had experienced at least one negative event related to their skin condition (entailing feelings of failure, humiliation or rejection) were eligible for this study. All participants would need to complete an online English-language writing activity; hence, internet access was a requirement. Participants were recruited from a range of sources including: first year undergraduate psychology students (*n* = 26) from Macquarie University, Sydney, who self-enrolled into this study for course credit; members of a skin condition-related Facebook group (*n* = 15) responding to a Facebook posting about the study, and patients at a Sydney-based dermatology clinic (*n* = 9) responding to invitational flyers posted in the clinic waiting room. Facebook and clinic participants received no compensation for study participation. A total of 50 eligible individuals provided informed consent and participated in the study (see Figure 1). An additional 22 individuals provided informed consent, but did not proceed with completing the study questionnaires, and were therefore excluded from analyses.

### Procedure

Following online informed consent, all participants completed the pre-writing questionnaire and were randomly and equally assigned using the Qualtrics randomiser function to either control or experimental writing conditions (both completed online) such that there were 25 participants in each condition; participants were blind to their condition allocation. Participants then immediately completed their assigned writing following specified instructions, and immediately thereafter completed a follow-up questionnaire (see Online Supplemental File for study protocol).

*Study Conditions: Experimental condition* was adapted from the MyCB self-compassion focused writing intervention (Przezdziecki et al., 2016; Sherman et al., 2018) developed for use with breast cancer survivors. In this instance, participants were first instructed to briefly introduce and describe in writing their deepest thoughts and emotions regarding a negative event they had experienced in relation to their visible skin condition. After this brief written introduction, MyCB participants were then provided with five specific self-compassion focused prompts to structure their writing based on the concept of self-compassion (Neff & Dahm, 2015). The writing was undertaken in the context of having a visible skin condition, and the prompts addressed treating one’s body with kindness, giving kind advice to the self, having connection with others who also experience body image difficulties, awareness of one’s circumstances and reactions in a broader context, and writing a self-compassionate letter to oneself. The *Control condition* entailed participants writing in an unstructured expressive manner about their deepest thoughts and emotions of a negative event they had experienced in relation to their skin condition (Pennebaker & Beall, 1986), commencing with a general description of topic (as per the Experimental condition) followed by general instructions to continue writing (‘Please describe the event further’). The control accounted for the potential effect of time taken to complete the writing task. Both writing conditions were allocated a maximum of 30 minutes writing time (see Online Supplemental File for writing prompts).

### Measures

**Self-compassion** before and after the writing tasks was measured with the 12-item Self-Compassion Scale Short Form (SCS-SF) (Raes, Pommier, Neff, & Van Gucht, 2011) assessing components of self-kindness, self-judgment, common humanity, isolation, mindfulness, and over-identification. Items such as ‘I try to be loving towards myself when I’m feeling emotional pain’ were rated on a 5-point scale (1 ‘almost never’ to 5 ‘almost always’) and a mean was calculated (range 1–5) with higher scores indicating greater self-compassion (Cronbach’s *α* = .90).

**Affect** before and after the writing tasks was measured with the 20-item Positive and Negative Affect Schedule (PANAS) consisting of the 10-item positive (Cronbach’s *α* = .84) and negative (Cronbach’s *α* = 82) affect subscales (Crawford & Henry, 2004; Watson, Clark, & Tellegen, 1988). Participants rated on a 5-point scale (1 ‘very slightly or not at all’ to 5 ‘extremely’) the extent to which they felt each of the 20 emotions in that present moment, such as ‘distressed’ or ‘proud’. Summed scores were obtained for each subscale (range 10–50).

**Body Image Disturbance** at study entry was treated as a covariate and measured with the 7-item Body Image Disturbance Questionnaire (Cash, Phillips, Santos, & Hrabosky, 2004). This scale was chosen as it assesses body-image specific distress rather than general psychological distress. The scale shares a positive relationship with depression, anxiety, and stress scores as measured by the Depression Anxiety and Stress Scale (Collison & Mahlberg, 2018). Items such as ‘do you ever avoid things because of your physical defect?’ were rated (1 ‘never’ to 5 ‘very often’) and higher mean scores indicated greater body image disturbance (*α* = .82; range 1–5).

**Perceived skin condition severity** participants’ subjective level of perceived severity of their skin condition was assessed with a single Likert-type scale ranging from 1 ‘Low Severity’ to 5 ‘High Severity’.

**Demographic and Medical Characteristics** documented pre-allocation included age, gender, education level, skin condition type, time since skin condition onset, whether treatment was received for the skin condition (yes/no). All demographic and medical characteristics and perceived condition severity were treated as potential covariates.

## Data analysis

All analyses were performed in SPSS version 25.0 (IBM Corp., 2017) unless otherwise specified. Significance was set at 0.05 for all tests. Descriptive analyses outlined sample characteristics. Chi-square and *t*-test analyses were used to identify covariates (i.e. testing for significant differences across pre-allocation outcome measures and sample characteristics between condition allocation and recruitment source – students vs. community). To quantify the extent of body image disturbance found in the present study, a two-sample t-test was conducted in the R statistics software (R Development Core Team, 2008) to compare the mean and standard deviation of body image disturbance found in our study to that of the sample on which the scale was originally validated. The R statistics software was required for this analysis as SPSS does not have the functionality for t-test comparisons based on summary data (i.e. means and standard deviations in absence of the raw data). Repeated measures ANCOVAs with mixed effects were then conducted for each follow-up outcome by study condition, controlling for the relevant identified covariates. Sample size was determined by the number of participants who self-enrolled into the study between March and September 2016.

### Ethics Statement

Institutional ethics approval was granted (Macquarie University HREC #5201600318; Trial Registration: ACTRN12618002025213).

## Results

Table 1 outlines pre-allocation sample characteristics and comparisons across condition allocation. The sample sources differed by gender, age, education, and perceived symptom severity and were therefore included as covariates in subsequent analyses. Mean body image distress scores in the skin condition sample were significantly higher in the current study (*M* = 2.40, *SD* = 0.98) than for the non-clinical sample on which the measure was originally validated (*M* = 1.70, *SD* = 0.96). This confirms the inclusion criterion that participants in this study with skin conditions were experiencing elevated body image distress (*t* = 4.52, *p* < .0005; Cash et al., 2004).

**Table 1.** Pre-allocation characteristics and bivariate comparisons.

	Whole Sample (*N* = 50)		Experimental (*N* = 25)		Control (*N* = 25)		*χ*^2^/*t*	*p*
	*n*	%	*n*	%	*n*	%		
Gender (Male)	15	28.80	8	32.00	7	28.00	0.10	.758
Education							3.71	.295
High School	27	51.90	12	48.00	13	52.00		
Vocation	6	11.50	5	20.00	1	4.00		
Undergraduate	9	17.30	3	12.00	6	24.00		
Postgraduate	10	19.20	5	20.00	5	20.00		
Skin Condition							2.87	.580
Acne	16	32.00	10	40.00	6	24.00		
Eczema	10	20.00	3	12.00	7	28.00		
Psoriasis	15	30.00	8	28.00	7	28.00		
Birthmark	4	8.00	2	8.00	2	8.00		
Other	5	10.00	8	12.00	7	12.00		
Time Since Diagnosis							2.09	.555
<6 months	2		1	4.00	1	4.00		
6 months to 1 year	1		1	4.00	0	0.00		
>1 year	47		23	92.00	24	96.00		
Receiving Tx^a^ (Yes)	25	48.10	13	52.00	11	44.00	0.32	.571
**Mean(*SD*)**								
Perceived Tx Efficacy^b^	1.17(0.82)		1.10(0.74)		1.15(0.90)		0.15	.879
Perceived Severity	3.00(1.16)		3.12(1.13)		2.84(1.21)		−0.84	.403
Age	27.13(10.78)		28.08(11.34)		26.72(10.63)		−0.44	.664
Self-Compassion	3.02(0.67)		2.85(0.65)		3.19(0.65)		−1.86	.069
BI disturbance^c^	2.40(0.98)		2.43(0.99)		2.38(0.99)		0.16	.871
Positive affect	29.98(8.64)		27.44(9.47)		32.52(7.02)		−2.16	.036
Negative affect	26.58(10.53)		28.52(10.53)		24.64(10.37)		1.31	.196

Note: Baseline means for outcomes differ to those reported in results as this table reports raw means and the results reports adjusted means. Tx = Treatment and BI = Body Image. ^a^Receiving treatment for skin condition. ^b^Perceived efficacy of treatment for skin condition. ^c^Body image disturbance.

**Figure 2.**
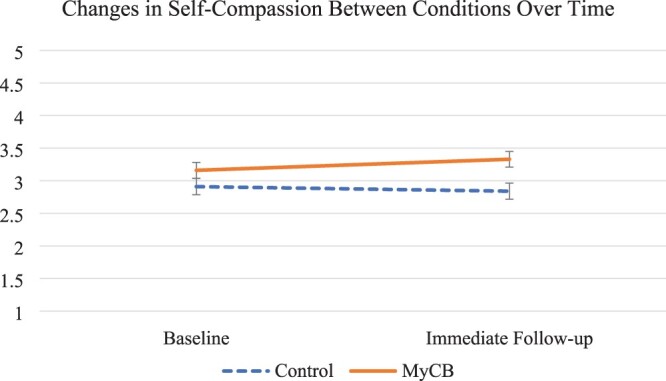
Change in self-compassion from baseline to immediate follow-up for both the intervention and control conditions. Error bars represent +/− 1SE around the mean.

**Figure 3.**
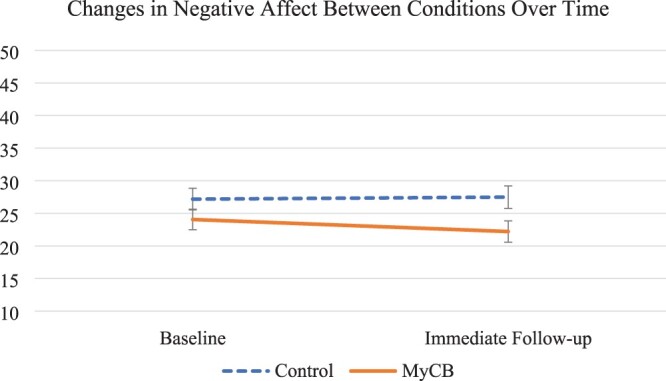
Change in negative affect from baseline to immediate follow-up for both the intervention and control conditions. Error bars represent +/− 1SE around the mean.

**Figure 4.**
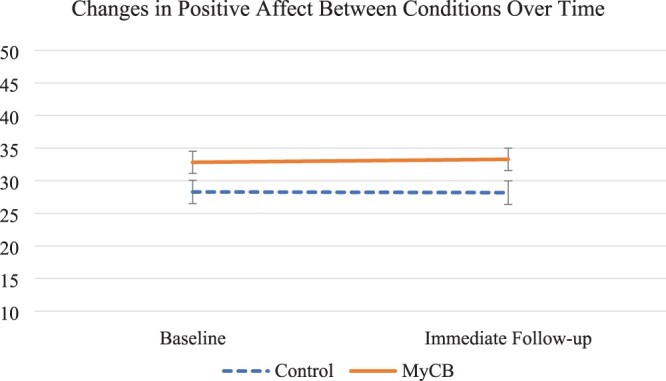
Change in positive affect from baseline to immediate follow-up for both the intervention and control conditions. Error bars represent +/− 1SE around the mean.

Regarding effects of the experimental allocation, repeated measure ANCOVA with mixed effects results revealed significant time by condition effects in self-compassion (*F* = 4.24, *p* = .046, $\eta_{partial}^{2}$= .09) and negative affect (*F* = 5.16, *p* = .028, $\eta_{partial}^{2}$= .11; see Table 2). Specifically, controlling for identified covariates, while there was no difference at baseline in self-compassion scores (control: *Mean* = 2.91, *SD* = 0.63; Intervention: Mean = 3.16, SD = 0.61; *F* = 2.16, *p* = .149) participants allocated to the experimental self-compassion writing condition reported significantly greater self-compassion at follow-up than control condition participants (control: *Mean* = 2.84, *SD* = 0.62; Intervention: *Mean* = 3.33, *SD* = 0.60; *F* = 8.52, *p* = .006). Controlling for covariates, there were also no baseline differences in negative affect (control: *Mean* = 27.18, *SD* = 8.30; Intervention: *Mean* = 24.06, *SD* = 7.90; *F* = 1.96, *p* = .169), similar to self-compassion, the experimental condition participants reported improved follow-up negative affect compared to controls (control: *Mean* = 27.49, *SD* = 8.63; Intervention: *Mean* = 22.21, *SD* = 8.20; *F* = 5.20, *p* = .028). There were no significant differences by condition in follow-up positive affect (see Figures 2–4 and Table 2).

**Table 2.** Comparisons between conditions over time on study outcomes controlling for relevant covariates.

	Baseline – *M*(*SD*)		Follow-up – *M*(*SD*)		*F*	*p*	$\eta_{partial}^{2}$
	Control	Intervention	Control	Intervention			
**Self-Compassion**					5.30	.026	0.11
Experimental Condition^a^	2.91(0.63)	3.16(0.61)	2.84(0.62)	3.33(0.60)	4.24	.046	0.09
Gender^b^					1.39	.245	0.03
Education					1.11	.298	0.03
Perceived Severity					0.02	.886	0.00
Age					2.65	.111	0.06
Body Image Disturbance					0.62	.434	0.01
**Negative Affect**					2.97	.092	0.07
Experimental Condition^a^	27.18(8.30)	24.06(7.90)	27.49(8.63)	22.21(8.20)	5.16	.028	0.11
Gender^b^					1.39	.245	0.03
Education					0.03	.033	0.00
Perceived Severity					0.60	.444	0.01
Age					3.58	.066	0.08
Body Image Disturbance					1.87	.178	0.04
**Positive Affect**					0.29	.591	0.01
Experimental Condition^a^	28.29(8.95)	32.83(8.53)	28.18(9.06)	33.29(8.63)	0.59	.447	0.01
Gender^b^					2.25	.141	0.14
Education					0.03	.873	0.00
Perceived Severity					1.35	.252	0.03
Age					0.24	.627	0.01
Body Image Disturbance					1.27	.265	0.03

^a^Reference = Controls.

^b^Reference = Female.

## Discussion

The aim of this study was to determine the feasibility of translating the evidence-based MyCB self-compassionate writing intervention to a health context other than breast cancer. Specifically, the efficacy of the MyCB intervention (Przezdziecki et al., 2016) for addressing body image concerns of breast cancer survivors has previously been demonstrated in a large randomised control (Sherman et al., 2018). In this feasibility study we utilised a pre-post experimental design and applied the MyCB writing intervention approach to individuals experiencing body image concerns arising from symptoms of a visible skin condition. This was compared to an active control condition who undertook an expressive writing activity regarding their body image concerns, without receiving the self-compassion prompts. This active control condition was a strength of the design as it accounts for time and attention in the writing task, ensuring that ensured that the study results reflected the self-compassion elements of writing, rather than general expressive writing. Study participants reported greater levels of body image disturbance than those in the original validation study of the body image disturbance questionnaire, confirming that the sample investigated in this study were generally experiencing elevated body image disturbance (Figure 4).

Following randomisation, participants in the MyCB writing condition reported significant improvements in self-compassion and negative affect compared with those in the expressive writing active control condition. These findings are consistent with that of the initial MyCB intervention feasibility study conducted within the breast cancer context (Przezdziecki & Sherman, 2016) and Leary et al. (Leary et al., 2007) who both experimentally manipulated self-compassion through writing. This reinforces the utility of a brief single dose intervention, such as the MyCB intervention, to improve negative affect and self-compassion in a different health context in which it was originally developed and trialled. Despite self-compassion theoretically having the capacity to enhance positive affect (Neff et al., 2018; Neff, Rude, & Kirkpatrick, 2007), there was no significant difference in positive affect between the experimental and control conditions in this pilot study, mirroring the findings of the prior MyCB intervention feasibility study undertaken in the breast cancer context (Przezdziecki & Sherman, 2016). Given that the large RCT demonstrated reductions in anxiety and depression that were maintained up to three months post-intervention (Sherman et al., 2018), it is possible that the impact of the MyCB intervention may be predominantly on ameliorating negative affect, rather than enhancing positive affect. Furthermore, it is possible that improvements in positive affect may take time to emerge and may not have been immediately detectable, given that this pilot study only measured outcomes immediately after participants engaged in the writing exercises. From a theoretical perspective, this pilot study adds to the growing body of literature (Braun et al., 2016; Sherman et al., 2018) supporting the use of self-compassion as a therapeutic intervention to address body image concerns.

The findings of this study need to be considered in light of the fact that this was a feasibility study. The sample size was relatively small, and there was only an immediate follow-up. Larger sample sizes would increase generalisability while longer follow-ups would ensure that the reported effects maintain over time, and may reveal delayed benefits of the intervention, such as possibly positive affect. A larger RCT of MyCB in this population could address these outcomes. It is also important to acknowledge that past research employing online interventions have had high drop-out rates, potentially leaving only highly motivated individuals in the study (Bessell, 2012; Bundy et al., 2013; Radtke, Ostergaard, Cooke, & Scholz, 2017; Richards & Richardson, 2012; Sundström et al., 2017; Williamson et al., 2015). This could potentially be biasing the results of online interventions. While we did not have a low drop out in this study, it is possible that only motivated individuals signed up. As such, future work should look at the efficacy of this intervention in a naturalistic setting.

Nevertheless, these findings have implications for the clinical management of body image concerns in individuals with visible skin conditions. MyCB is a private, single dose, low cost writing intervention that can be self-administered anywhere via the internet independent of clinical support. As such, patients with visible skin conditions would benefit from being directed towards self-compassionate writing exercises, such as MyCB, in conjunction with standard care. This would not only increase the time available for clinicians to work on treating the skin condition, but also help to alleviate some of the negative affect that one might experience as a result of body image concerns related to the skin condition.

## Supplementary Material

Supplemental Material

## References

[CIT0001] Ahmed, A., Steed, L., Burden-Teh, E., Shah, R., Sanyal, S., Tour, S., … Bewley, A. (2018). Identifying key components for a psychological intervention for people with vitiligo–a quantitative and qualitative study in the United Kingdom using web-based questionnaires of people with vitiligo and healthcare professionals. *Journal of the European Academy of Dermatology and Venereology*, 1–9. doi: 10.1111/jdv.15168 29972710

[CIT0002] Albertson, E. R., Neff, K. D., & Dill-Shackleford, K. E. (2015). Self-compassion and body dissatisfaction in women: A randomized controlled trial of a brief meditation intervention. *Mindfulness*, *6*(3), 444–454.

[CIT0003] Allen, A. B., & Leary, M. R. (2010). Self-compassion, stress, and coping. *Social and Personality Psychology Compass*, *4*(2), 107–118. doi: 10.1111/j.1751-9004.2009.00245.x 20686629 PMC2914331

[CIT0004] Arch, J. J., Brown, K. W., Dean, D. J., Landy, L. N., Brown, K. D., & Laudenslager, M. L. (2014). Self-compassion training modulates alpha-amylase, heart rate variability, and subjective responses to social evaluative threat in women. *Psychoneuroendocrinology*, *42*, 49–58. 24636501 10.1016/j.psyneuen.2013.12.018 PMC3985278

[CIT0005] Atkinson, M. J., & Wade, T. D. (2015). Mindfulness-based prevention for eating disorders: A school-based cluster randomized controlled study. *International Journal of Eating Disorders*, *48*(7), 1024–1037. 10.1002/eat.22416 26052831

[CIT0006] Atkinson, M. J., & Wade, T. D. (2016). Does mindfulness have potential in eating disorders prevention? A preliminary controlled trial with young adult women. *Early Intervention in Psychiatry*, *10*(3), 234–245. 24894735 10.1111/eip.12160

[CIT0007] Australian Bureau of Statistics. (2018). Household Use of Information Technology, Australia, 2016–17. (8146.0).

[CIT0008] Baker, C. S., Foley, P. A., & Braue, A. (2013). Psoriasis uncovered–measuring burden of disease impact in a survey of Australians with psoriasis. *Australasian Journal of Dermatology*, *54*(S1), 1–6. doi: 10.1111/ajd.12010 23379483

[CIT0009] Beatty, L., Kemp, E., Binnion, C., Turner, J., Milne, D., Butow, P., … Koczwara, B. (2017). Uptake and adherence to an online intervention for cancer-related distress: Older age is not a barrier to adherence but may be a barrier to uptake. *Supportive Care in Cancer*, *25*(6), 1905–1914. 28155018 10.1007/s00520-017-3591-1

[CIT0010] Bessell, A. (2012). Computer-based psychosocial interventions. *The Oxford Handbook of the Psychology of Appearance* (chap. 39), 568–580.

[CIT0011] Braun, T. D., Park, C. L., & Gorin, A. (2016). Self-compassion, body image, and disordered eating: A review of the literature. *Body Image*, *17*, 117–131. 27038782 10.1016/j.bodyim.2016.03.003

[CIT0012] Bundy, C., Pinder, B., Bucci, S., Reeves, D., Griffiths, C. E. M., & Tarrier, N. (2013). A novel, web-based, psychological intervention for people with psoriasis: The electronic Targeted Intervention for Psoriasis (eTIPs) study. *British Journal of Dermatology*, *169*(2), 329–336. doi: 10.1111/bjd.12350 23551271

[CIT0013] Cash, T. F. (2011). Cognitive-behavioral perspectives on body image. In T. F. Cash & L. Smolak (Eds.), *Body image: A handbook of science, practice, and prevention* (pp. 39–47). New York: Guilford Press.

[CIT0014] Cash, T. F., Phillips, K. A., Santos, M. T., & Hrabosky, J. I. (2004). Measuring “negative body image”: Validation of the body image disturbance questionnaire in a nonclinical population. *Body Image*, *1*(4), 363–372. doi: 10.1016/j.bodyim.2004.10.001

[CIT0015] Cash, T. F., & Szymanski, M. L. (1995). The development and validation of the body-image ideals questionnaire. *Journal of Personality Assessment*, *64*(3), 466–477. doi: 10.1207/s15327752jpa6403_6 16367722

[CIT0016] Collison, J., & Mahlberg, J. (2018). Factor analysis and psychometric validation of the body image disturbance questionnaire in an Australian undergraduate sample. *Australian Psychologist*, *53*(3), 195–202.

[CIT0017] Consedine, N. S., Krivoshekova, Y. S., & Harris, C. R. (2007). Bodily embarrassment and judgment concern as separable factors in the measurement of medical embarrassment: Psychometric development and links to treatment-seeking outcomes. *British Journal of Health Psychology*, *12*(3), 439–462. doi: 10.1348/135910706X118747 17640455

[CIT0018] Costa, J., & Pinto-Gouveia, J. (2013). Experiential avoidance and self-compassion in chronic pain. *Journal of Applied Social Psychology*, *43*(8), 1578–1591.

[CIT0019] Crawford, J. R., & Henry, J. D. (2004). The positive and negative affect schedule (PANAS): Construct validity, measurement properties and normative data in a large non-clinical sample. *British Journal of Clinical Psychology*, *43*(3), 245–265. 10.1348/0144665031752934 15333231

[CIT0020] Duarte, C., Ferreira, C., Trindade, I. A., & Pinto-Gouveia, J. (2015). Body image and college women’s quality of life: The importance of being self-compassionate. *Journal of Health Psychology*, *20*(6), 754–764. 26032792 10.1177/1359105315573438

[CIT0021] Finlay-Jones, A., Kane, R., & Rees, C. (2017). Self-compassion online: A pilot study of an internet-based self-compassion cultivation program for psychology trainees. *Journal of Clinical Psychology*, *73*(7), 797–816. 27787877 10.1002/jclp.22375

[CIT0022] Finlay-Jones, A. L., Rees, C. S., Kane, R. T., & van der Feltz-Cornelis, C. (2015). Self-compassion, emotion regulation and stress among Australian psychologists: Testing an emotion regulation model of self-compassion using structural equation modeling. *PloS one*, *10*(7), e0133481. 26207900 10.1371/journal.pone.0133481 PMC4514830

[CIT0023] Ginsburg, I. H., & Link, B. G. (1993). Psychosocial consequences of rejection and stigma feelings in psoriasis patients. *International Journal of Dermatology*, *32*(8), 587–591. doi: 10.1111/j.1365-4362.1993.tb05031.x 8407075

[CIT0024] Gratz, K. L., & Roemer, L. (2004). Multidimensional assessment of emotion regulation and dysregulation: Development, factor structure, and initial validation of the difficulties in emotion regulation scale. *Journal of Psychopathology and Behavioral Assessment*, *26*(1), 41–54.

[CIT0025] Gupta, M. A., & Gupta, A. K. (1998). Depression and suicidal ideation in dermatology patients with acne, alopecia areata, atopic dermatitis and psoriasis. *British Journal of Dermatology*, *139*(5), 846–850. doi: 10.1046/j.1365-2133.1998.02511.x 9892952

[CIT0026] Gupta, M. A., & Gupta, A. K. (2013). Cutaneous body image dissatisfaction and suicidal ideation: Mediation by interpersonal sensitivity. *Journal of Psychosomatic Research*, *75*(1), 55–59. doi: 10.1016/j.jpsychores.2013.01.015 23751239

[CIT0027] Gupta, M. A., Gupta, A. K., & Knapp, K. (2015). Dissatisfaction with cutaneous body image is directly correlated with insomnia severity: A prospective study in a non-clinical sample. *Journal of Dermatological Treatment*, *26*(2), 193–197. doi: 10.3109/09546634.2014.883060 24511911

[CIT0028] Hinkley, S. B. (2015). *Not just “skin deep”: understanding cutaneous body image in community and clinical samples*. Dallas: The University of Texas.

[CIT0029] Hong, J., Koo, B., & Koo, J. (2008). The psychosocial and occupational impact of chronic skin disease. *Dermatologic Therapy*, *21*(1), 54–59. doi: 10.1111/j.1529-8019.2008.00170.x 18318886

[CIT0030] IBM Corp. (2017). *IBM SPSS statistics for windows (version 25.0)*. Armonk, NY: IBM Corp.

[CIT0031] Karyotaki, E., Riper, H., Twisk, J., Hoogendoorn, A., Kleiboer, A., Mira, A., … Cuijpers, P. (2017). Efficacy of self-guided internet-based cognitive behavioral therapy in the treatment of depressive symptoms: A meta-analysis of individual participant data. *JAMA Psychiatry*, *74*(4), 351–359. 28241179 10.1001/jamapsychiatry.2017.0044

[CIT0032] Kellett, S. (2014). Shame-fused acne: A biopsychosocial conceptualisation and treatment rationale. In P. Gilbert, & J. Miles (Eds.), *Body shame* (pp. 149–168). London: Routledge.

[CIT0033] Kelly, A. C., Zuroff, D. C., & Shapira, L. B. (2009). Soothing oneself and resisting self-attacks: The treatment of two intrapersonal deficits in depression vulnerability. *Cognitive Therapy and Research*, *33*(3), 301–313. doi: 10.1007/s10608-008-9202-1

[CIT0034] Köhle, N., Drossaert, C. H., Schreurs, K. M., Hagedoorn, M., Verdonck-de Leeuw, I. M., & Bohlmeijer, E. T. (2015). A web-based self-help intervention for partners of cancer patients based on acceptance and commitment therapy: A protocol of a randomized controlled trial. *BMC Public Health*, *15*(1), 1471–2458. 10.1186/s12889-015-1656-y PMC4392862 25884187

[CIT0035] Kouris, A., Platsidaki, E., Kouskoukis, C., & Christodoulou, C. (2017). Psychological parameters of psoriasis. *Psychiatriki*, *28*(1), 54–59. 28541239 10.22365/jpsych.2017.281.54

[CIT0036] Krieger, T., Altenstein, D., Baettig, I., Doerig, N., & Holtforth, M. G. (2013). Self-compassion in depression: Associations with depressive symptoms, rumination, and avoidance in depressed outpatients. *Behavior Therapy*, *44*(3), 501–513. 23768676 10.1016/j.beth.2013.04.004

[CIT0037] Kuijpers, W., Groen, W. G., Aaronson, N. K., & van Harten, W. H. (2013). A systematic review of web-based interventions for patient empowerment and physical activity in chronic diseases: Relevance for cancer survivors. *Journal of Medical Internet Research*, *15*(2), e37. 23425685 10.2196/jmir.2281 PMC3636300

[CIT0038] Law, M., Chuh, A., Lee, A., & Molinari, N. (2010). Acne prevalence and beyond: Acne disability and its predictive factors among Chinese late adolescents in Hong Kong. *Clinical and Experimental Dermatology*, *35*(1), 16–21. doi: 10.1111/j.1365-2230.2009.03340.x 19486044

[CIT0039] Leary, M. R., Tate, E. B., Adams, C. E., Batts Allen, A., & Hancock, J. (2007). Self-compassion and reactions to unpleasant self-relevant events: The implications of treating oneself kindly. *Journal of Personality and Social Psychology*, *92*(5), 887–904. doi: 10.103/0022-3514.92.5.887 17484611

[CIT0040] MacBeth, A., & Gumley, A. (2012). Exploring compassion: A meta-analysis of the association between self-compassion and psychopathology. *Clinical Psychology Review*, *32*(6), 545–552. doi: 10.1016/j.cpr.2012.06.003 22796446

[CIT0041] Melioli, T., Bauer, S., Franko, D. L., Moessner, M., Ozer, F., Chabrol, H., & Rodgers, R. F. (2016). Reducing eating disorder symptoms and risk factors using the internet: A meta-analytic review. *International Journal of Eating Disorders*, *49*(1), 19–31. 10.1002/eat.22477 26607683

[CIT0042] Merz, E. L., Fox, R. S., & Malcarne, V. L. (2014). Expressive writing interventions in cancer patients: A systematic review. *Health Psychology Review*, *8*(3), 339–361. 25053218 10.1080/17437199.2014.882007

[CIT0043] Murray, C. D., & Rhodes, K. (2005). Nobody likes damaged goods: The experience of adult visible acne. *British Journal of Health Psychology*, *10*(2), 183–202. doi: 10.1348/135910705X26128 15969849

[CIT0044] Neff, K. D. (2003). The development and validation of a scale to measure self-compassion. *Self and Identity*, *2*(3), 223–250. doi: 10.1080/15298860309027

[CIT0045] Neff, K. D., & Dahm, K. A. (2015). Self-compassion: What it is, what it does, and how it relates to mindfulness. In B. D. Ostafin, M. D. Robinson, & B. P. Meier (Eds.), *Handbook of mindfulness and self-regulation* (pp. 121–137). New York: Springer.

[CIT0046] Neff, K. D., & Germer, C. (2017). Self-compassion and psychological well-being. In E. M. Seppala, E. Simon-Thomas, S. L. Brown, M. C. Worline, C. D. Cameron, & J. R. Doty (Eds.), *The Oxford handbook of compassion science* (Chap. 27, pp. 371). Oxford: Oxford University Press.

[CIT0047] Neff, K. D., & Germer, C. K. (2013). A pilot study and randomized controlled trial of the mindful self-compassion program. *Journal of Clinical Psychology*, *69*(1), 28–44. 23070875 10.1002/jclp.21923

[CIT0048] Neff, K. D., Kirkpatrick, K. L., & Rude, S. S. (2007). Self-compassion and adaptive psychological functioning. *Journal of Research in Personality*, *41*(1), 139–154. doi: 10.1016/j.jrp.2006.03.004

[CIT0049] Neff, K. D., Long, P., Knox, M. C., Davidson, O., Kuchar, A., Costigan, A., … Breines, J. G. (2018). The forest and the trees: Examining the association of self-compassion and its positive and negative components with psychological functioning. *Self and Identity*, *17*(6), 627–645.

[CIT0050] Neff, K. D., Rude, S. S., & Kirkpatrick, K. L. (2007). An examination of self-compassion in relation to positive psychological functioning and personality traits. *Journal of Research in Personality*, *41*(4), 908–916. doi: 10.1016/j.jrp.2006.08.002

[CIT0051] Odou, N., & Brinker, J. (2015). Self-compassion, a better alternative to rumination than distraction as a response to negative mood. *The Journal of Positive Psychology*, *10*(5), 447–457.

[CIT0052] Pennebaker, J. W. (1997). Writing about emotional experiences as a therapeutic process. *Psychological Science*, *8*(3), 162–166. doi: 10.1111/j.1467-9280.1997.tb00403.x

[CIT0053] Pennebaker, J. W., & Beall, S. K. (1986). Confronting a traumatic event: Toward an understanding of inhibition and disease. *Journal of Abnormal Psychology*, *95*(3), 274–281. doi: 10.1037//0021-843X.95.3.274 3745650

[CIT0054] Picardi, A., Mazzotti, E., & Pasquini, P. (2006). Prevalence and correlates of suicidal ideation among patients with skin disease. *Journal of the American Academy of Dermatology*, *54*(3), 420–426. doi: 10.1016/j.jaad.2005.11.1103 16488292

[CIT0055] Pinto-Gouveia, J., Duarte, C., Matos, M., & Fráguas, S. (2014). The protective role of self-compassion in relation to psychopathology symptoms and quality of life in chronic and in cancer patients. *Clinical Psychology & Psychotherapy*, *21*(4), 311–323. 23526623 10.1002/cpp.1838

[CIT0056] Postel, M. G., de Haan, H. A., Ter Huurne, E. D., Becker, E. S., & de Jong, C. A. (2010). Effectiveness of a web-based intervention for problem drinkers and reasons for dropout: Randomized controlled trial. *Journal of Medical Internet Research*, *12*(4), e68. 21163776 10.2196/jmir.1642 PMC3056532

[CIT0057] Przezdziecki, A., Alcorso, J., & Sherman, K. A. (2016). My Changed Body: Background, development and acceptability of a self-compassion based writing activity for female survivors of breast cancer. *Patient Education and Counseling*, *99*(5), 870–874. doi: 10.1016/j.pec.2015.12.011 26754617

[CIT0058] Przezdziecki, A., & Sherman, K. A. (2016). Modifying affective and cognitive responses regarding body image difficulties in breast cancer survivors using a self-compassion-based writing intervention. *Mindfulness*, *7*(5), 1142–1155. doi: 10.1007/s12671-016-0557-1

[CIT0059] Przezdziecki, A., Sherman, K. A., Baillie, A., Taylor, A., Foley, E., & Stalgis-Bilinski, K. (2013). My changed body: Breast cancer, body image, distress and self-compassion. *Psycho-Oncology*, *22*(8), 1872–1879. doi: 10.1002/pon.3230 23203842

[CIT0060] Radtke, T., Ostergaard, M., Cooke, R., & Scholz, U. (2017). Web-based alcohol intervention: Study of systematic attrition of heavy drinkers. *Journal of Medical Internet Research*, *19*(6), e217. 28659251 10.2196/jmir.6780 PMC5508117

[CIT0061] Raes, F. (2010). Rumination and worry as mediators of the relationship between self-compassion and depression and anxiety. *Personality and Individual Differences*, *48*(6), 757–761.

[CIT0062] Raes, F., Pommier, E., Neff, K. D., & Van Gucht, D. (2011). Construction and factorial validation of a short form of the self-compassion scale. *Clinical Psychology and Psychotherapy*, *18*(3), 250–255. doi: 10.1002/cpp.702 21584907

[CIT0063] R Development Core Team. (2008). *R: A language and environment for statistical computing*. Vienna: R Foundation for Statistical Computing.

[CIT0064] Richards, J. M., Beal, W. E., Seagal, J. D., & Pennebaker, J. W. (2000). Effects of disclosure of traumatic events on illness behavior among psychiatric prison inmates. *Journal of Abnormal Psychology*, *109*(1), 156–160. 10740948 10.1037//0021-843x.109.1.156

[CIT0065] Richards, D., & Richardson, T. (2012). Computer-based psychological treatments for depression: A systematic review and meta-analysis. *Clinical Psychology Review*, *32*(4), 329–342. 22466510 10.1016/j.cpr.2012.02.004

[CIT0066] Rodgers, R. F., Donovan, E., Cousineau, T., Yates, K., McGowan, K., Cook, E. … Franko, D. L. (2018). Bodimojo: Efficacy of a mobile-based intervention in improving body image and self-compassion among adolescents. *Journal of Youth and Adolescence*, *47*, 1363–1372. 29349593 10.1007/s10964-017-0804-3

[CIT0067] Roemer, L., Lee, J. K., Salters-Pedneault, K., Erisman, S. M., Orsillo, S. M., & Mennin, D. S. (2009). Mindfulness and emotion regulation difficulties in generalized anxiety disorder: Preliminary evidence for independent and overlapping contributions. *Behavior Therapy*, *40*(2), 142–154. 19433145 10.1016/j.beth.2008.04.001 PMC3719394

[CIT0068] Rumsey, N. (2018). Psychosocial adjustment to skin conditions resulting in visible difference (disfigurement): What do we know? Why don’t we know more? How shall we move forward? *International Journal of Women’s Dermatology*, *4*(1), 2–7. doi: 10.1016/j.ijwd.2017.09.005 PMC5986108 29872669

[CIT0069] Sarwer, D., & Spitzer, J. (2015). Cosmetic surgical procedures of the body. In T. F. Cash (Ed.), *Encyclopedia of body image and human appearance* (Vol. 1, pp. 360–365). San Diego, CA: Academic Press.

[CIT0070] Shaw, L. K., Sherman, K., Fitness, J., Elder, E., & Breast Cancer Network Australia. (2018). Factors associated with romantic relationship formation difficulties in women with breast cancer. *Psycho-Oncology*, *27*(4), 1270–1276. 29430771 10.1002/pon.4666

[CIT0071] Shenefelt, P. D. (2010). Psychological interventions in the management of common skin conditions. *Psychology Research and Behavior Management*, *3*, 51–63. 22110329 10.2147/prbm.s7072 PMC3218765

[CIT0072] Sherman, K. A., Przezdziecki, A., Alcorso, J., Kilby, C. J., Elder, E., Boyages, J., … Mackie, H. (2018). Reducing body image–related distress in women with breast cancer using a structured online writing exercise: Results from the my changed body randomized controlled trial. *Journal of Clinical Oncology*, *36*(19), 1930–1940. doi: 10.1200/jco.2017.76.3318 29688834

[CIT0073] Smyth, J. M. (1998). Written emotional expression: Effect sizes, outcome types, and moderating variables. *Journal of Consulting and Clinical Psychology*, *66*(1), 174–184. doi:10.1037//0022-006X.66.1.174 9489272

[CIT0074] Stanton, A. L., Danoff-Burg, S., Sworowski, L. A., Collins, C. A., Branstetter, A. D., Rodriguez-Hanley, A., … Austenfeld, J. L. (2002). Randomized, controlled trial of written emotional expression and benefit finding in breast cancer patients. *Journal of Clinical Oncology*, *20*(20), 4160–4168. doi: 10.1220/JCO.2002.08.521 12377959

[CIT0075] Sundström, C., Blankers, M., & Khadjesari, Z. (2017). Computer-based interventions for problematic alcohol use: A review of systematic reviews. *International Journal of Behavioral Medicine*, *24*(5), 646–658. 27757844 10.1007/s12529-016-9601-8 PMC5608865

[CIT0076] Van Dam, N. T., Sheppard, S. C., Forsyth, J. P., & Earleywine, M. (2011). Self-compassion is a better predictor than mindfulness of symptom severity and quality of life in mixed anxiety and depression. *Journal of Anxiety Disorders*, *25*(1), 123–130. doi: 10.1016/j.janxdis.2010.08.011 20832990

[CIT0077] Watson, D., Clark, L. A., & Tellegen, A. (1988). Development and validation of brief measures of positive and negative affect: The PANAS scales. *Journal of Personality and Social Psychology*, *54*(6), 1063–1070. 3397865 10.1037//0022-3514.54.6.1063

[CIT0078] White, C. A. (2000). Body image dimensions and cancer: A heuristic cognitive behavioural model. *Psycho-Oncology*, *9*(3), 183–192. doi: 10.1002/1099-1611 10871714

[CIT0079] Williamson, H., Griffiths, C., & Harcourt, D. (2015). Developing young person’s face IT: Online psychosocial support for adolescents struggling with conditions or injuries affecting their appearance. *Health Psychology Open*, *July–December*, 1–12. doi:10.1177/2055102915619092 PMC5193303 28070380

