# Supplementary material for: Enhancing self-compassion in individuals with visible skin conditions: randomised pilot of the ‘My Changed Body’ self-compassion writing intervention
Source: Health Psychol Behav Med. 2019 Mar 18;7(1):62–77. doi: 10.1080/21642850.2019.1587298 (PMC8114345; doi:10.1080/21642850.2019.1587298)
Supplement: Supplemental Material [file RHPB_A_1587298_SM4312.docx]

**Enhancing Self-Compassion in Individuals with Visible Skin Conditions: Randomised Pilot of the ‘My Changed Body’ Self-Compassion Writing Intervention**

**Study Protocol**

Kerry Anne Sherman^1^, [kerry.sherman@mq.edu.au](mailto:kerry.sherman@mq.edu.au), ORCiD: 0000-0001-7780-6668

Tegan Roper^1^,

Christopher Jon Kilby^1^, [Christopher.kilby@mq.edu.au](mailto:Christopher.kilby@mq.edu.au), ORCiD: 0000-0002-5783-6525

^1^ Affiliation: Centre for Emotional Health, Department of Psychology, Macquarie University, Sydney, Australia

Postal address: 4 Second Way, Macquarie University, Sydney, Australia

Phone number: (+612) 9762 5302

Twitter Handles: @PsyMQ, @MacquarieUniCEH

Facebook: @PsyMQ, @MacquarieUniCEH

Study Design

A randomised pilot was used to assess the efficacy of an online self-compassion based expressive writing task with specific writing prompts – *My Changed Body* (MyCB) – to improve self-compassion and affect in male and female participants with a visible skin condition (e.g., eczema, psoriasis, acne). MyCB was compared to an online expressive writing task which did not include the specific self-compassion based writing prompts. Ethics approval was granted by the Macquarie University Human Research Ethics Committee (University HREC No: 5201600318).

Participants

Participants were recruited from March 2016 to September 2016 and were eligible to participate if they self-identified as being a) over 18 years of age, b) experiencing a visible skin condition, c) had experienced at least one negative event related to their skin condition, and d) had access to the internet. A total of 50 eligible individuals provided informed consent and participated in the study. Participants were recruited from either Macquarie University first-year psychology students (*n*=26), a skin condition-related Facebook group (*n*=15), or a Sydney-based dermatology clinic (*n*=9). University students and Facebook users responded to an electronic advertisement. Participants from the dermatology clinic responded to a study flyer posted in the clinic waiting room.

Procedure

Following online registration and online consent, all participants completed the baseline questionnaire. Participants were then randomly allocated to either the MyCB Intervention group or the expressive writing control condition via the computer using the Qualtrics randomizer function. Participants were blinded to condition allocation. Immediately after randomization, participants completed their assigned writing task. Immediately after completing the writing task, participants completed the follow-up questionnaire. Participants had to complete all sections of the writing task to proceed to the follow-up survey. Those choosing not to complete the writing task could withdraw from the study by closing their internet browser, thus ending their participation.

Schema and treatment plan: Not applicable

Rules for dose modification: Not applicable

Intervention

Participants in the intervention condition received immediate access to MyCB expressive writing task with specific writing prompt after completing the baseline questionnaire. MyCB assists users in perceiving their body and visible skin condition in a caring and supportive way, by allowing them to describe their deepest thoughts and emotions. MyCB starts with a general description of a negative event related to changes in their body. This is proceeded by five specific prompts (totalling in 6 prompts) to structure their writing by beginning with a narrow focus on the negative aspects of the self, and gradually writing in a more self-compassionate perspective (i.e., considering other women’s perspectives), and finally ending with a narrow focus on the personal situation (see Table 1 for intervention prompts). Prompts were presented in separate text boxes to assist in organising the participants writing. MyCB is purposefully brief to enhance the acceptability of the task, reduce time burden, and maximise the user-friendly aspects of the activity. The whole intervention takes approximately 30 minutes to complete.

Participants allocated to the active control condition also completed an online expressive writing task that was matched with the intervention for the number of text boxes and time of completed. The control condition received the same initial prompt to write about a negative body image-related event but without any self-compassionate writing prompts. Following text boxes included the prompt “Please describe the event further” and were absent of any specific writing prompts. The sixth and final text box asked participants to provide a conclusion to their event. This control condition ensured that the effect of writing or thinking about a negative event related to one’s body image, and the effect of time taken to complete the task of writing was accounted for (see Table 2 for control prompts).

**Table 1.** *My Changed Body* writing prompts

| Writing activity task | Prompt |
| --- | --- |
| 1. Introduction | Think about a negative event that you have experienced regarding symptoms of your skin condition that made you feel bad about yourself – something that involved sadness, embarrassment, failure, or rejection. Please set the scene and write an introduction for the negative event in the space below: |
| 1. Kindness to your body | Think about how your body has changed through your experience with your skin condition. Please write a paragraph about whether or not you have treated your body and yourself with kindness during this time. |
| 1. Kind advice | Keeping in mind how your body has changed as a result of having a skin condition, please write a paragraph showing understanding, kindness, and concern for yourself in a way you would help a friend who had undergone the experience. |
| 1. Connection with others | Please write a paragraph about ways in which other people also experience similar events (i.e., how other people may be feeling uncomfortable about their appearance or body). |

**Table 1 (cont).** *My Changed Body* writing prompts.

| Writing activity task | Prompt |
| --- | --- |
| 1. Awareness of the bigger picture | Please look at your feelings using a bigger perspective of the situation. See if you can put some space between the event and your reactions. Think about all the issues that have contributed and write about your feelings using this outlook. |
| 1. Conclusion – A letter to myself | Write a self-compassionate letter to yourself, perhaps extending this to a letter to all people with skin conditions. Having a skin condition, what are some of the things you’ve needed to hear about your body? What are you needing to say to yourself? What would you say to all people who are struggling with these issues? |

**Table 2.** Control condition writing prompts

| Writing activity task | Prompt |
| --- | --- |
| 1. Introduction | Please set the scene and write an introduction for the negative event in the space below: |
| 1. Part 2 | Please describe the event further |
| 1. Part 3 | Please describe the event further |
| 1. Part 4 | Please describe the event further |
| 1. Part 5 | Please describe the event further |
| 1. Part 6 | Please write a conclusion about the event |

Measurement of Treatment Effect

*Primary Outcomes*

Self-compassion (immediately before and following the writing task) – Measured with the 12-item Self-compassion Scale – Short Form (Raes, Pommier, Neff, & Van Gucht, 2011). A mean score was calculated, with scores ranging from 1 to 5 with higher scores indicative of higher self-compassion.

Positive and Negative Affect (immediately before and following the writing task) – Measured with the 20-item Positive and Negative Affect Schedule consisting of a 10-item momentary positive affect subscale and a 10-item momentary negative affect subscale. Scores ranged from 10 to 50 on both subscales with higher scores on each subscale representing higher endorsement of the subscales affect.

*Covariates*

A range of potential covariates were measured immediately before the writing task including demographics and medical history (age, gender, education level, skin condition type, time since skin condition onset, recipient of treatment [yes/no], perceived skin severity [from 1 “Low Severity” to 5 “High Severity”]) and body image distress (measured via the 7-item body image disturbance questionnaire, higher mean scores [range 1-5] represent greater body image disturbance; Cash, Phillips, Santos, & Hrabosky, 2004).

Analysis

*Study Objectives and Statistical Analyses*

The primary objective was to assess whether usual care plus access to MyCB may be a possible approach to promote self-compassion in a population of individuals with visible skin conditions. This work builds from prior work with MyCB which has demonstrated that MyCB produces immediate increases in self-compassion that lead to later improvements in body image disturbance in a sample of breast cancer survivors (Sherman et al., 2018). If this pilot demonstrates that MyCB does result in immediate shifts in self-compassion, then a larger scale longitudinal randomised controlled trial would be justified to explore the possible benefits of MyCB on body image disturbance in this population.

All analyses were performed in SPSS version 25.0 (IBM Corp., 2017) unless otherwise specified. Significance was set at 0.05 for all tests. Descriptive analyses outlined sample characteristics. Chi-square and *t*-test analyses were used to identify covariates (i.e., testing for significant differences across pre-allocation outcome measures and sample characteristics between condition allocation and recruitment source - students vs. community). To quantify the extent of body image disturbance found in the present study, a two-sample t-test was conducted in the R statistics software (R Development Core Team, 2008) to compare the mean and standard deviation of body image disturbance found in our study to that of the sample on which the scale was originally validated. The R statistics software was required for this analysis as SPSS does not have the functionality for t-test comparisons based on summary data (i.e., means and standard deviations in absence of the raw data). Repeated measures ANCOVAs with mixed effects were then conducted for each follow-up outcome by study condition, controlling for the relevant identified covariates. Sample size was determined by the number of participants who self-enrolled into the study between March to September 2016.

**References**

Cash, T. F., Phillips, K. A., Santos, M. T., & Hrabosky, J. I. (2004). Measuring “negative body image”: Validation of the Body Image Disturbance Questionnaire in a nonclinical population. *Body Image, 1*(4), pp. 363-372. doi:10.1016/j.bodyim.2004.10.001

IBM Corp. (2017). IBM SPSS Statistics for Windows (Version 25.0). Armonk, NY: IBM Corp.

R Development Core Team. (2008). R: A language and environment for statistical computing. Vienna, Austria: R Foundation for Statistical Computing.

Raes, F., Pommier, E., Neff, K. D., & Van Gucht, D. (2011). Construction and factorial validation of a short form of the self‐compassion scale. *Clinical Psychology and Psychotherapy, 18*(3), pp. 250-255. doi:10.1002/cpp.702

Sherman, K. A., Przezdziecki, A., Alcorso, J., Kilby, C. J., Elder, E., Boyages, J., . . . Mackie, H. (2018). Reducing Body Image–Related Distress in Women With Breast Cancer Using a Structured Online Writing Exercise: Results From the My Changed Body Randomized Controlled Trial. *Journal of Clinical Oncology, 36*(19), pp. 1930-1940. doi:10.1200/jco.2017.76.3318
